# Adenoviruses Using the Cancer Marker EphA2 as a Receptor In Vitro and In Vivo by Genetic Ligand Insertion into Different Capsid Scaffolds

**DOI:** 10.1371/journal.pone.0095723

**Published:** 2014-04-23

**Authors:** Michael Behr, Johanna K. Kaufmann, Patrick Ketzer, Sarah Engelhardt, Martin Mück-Häusl, Pamela M. Okun, Gabriele Petersen, Frank Neipel, Jessica C. Hassel, Anja Ehrhardt, Alexander H. Enk, Dirk M. Nettelbeck

**Affiliations:** 1 Oncolytic Adenovirus Group, German Cancer Research Center (DKFZ), Heidelberg, Germany; 2 Max von Pettenkofer-Institute, Department of Virology, Ludwig-Maximilians-University Munich, Munich, Germany; 3 Department of General Pediatrics, Division of Inherited Metabolic Diseases, Heidelberg University Hospital, Heidelberg, Germany; 4 COS, CellNetworks Deep Sequencing Core Facility, University Heidelberg, Heidelberg, Germany; 5 Institute for Clinical and Molecular Virology, Erlangen University Hospital, Erlangen, Germany; 6 Department of Dermatology, Heidelberg University Hospital, Heidelberg, Germany; 7 Institute of Virology and Microbiology, Center for Biomedical Education and Research, Department of Human Medicine, Faculty of Health, University Witten/Herdecke, Witten, Germany; Swedish Medical Center, United States of America

## Abstract

Adenoviral gene therapy and oncolysis would critically benefit from targeted cell entry by genetically modified capsids. This requires both the ablation of native adenovirus tropism and the identification of ligands that remain functional in virus context. Here, we establish cell type-specific entry of HAdV-5-based vectors by genetic ligand insertion into a chimeric fiber with shaft and knob domains of the short HAdV-41 fiber (Ad5T/41sSK). This fiber format was reported to ablate transduction *in vitro* and biodistribution to the liver *in vivo*. We show that the YSA peptide, binding to the pan-cancer marker EphA2, can be inserted into three positions of the chimeric fiber, resulting in strong transduction of EphA2-positive but not EphA2-negative cells of human melanoma biopsies and of tumor xenografts after intratumoral injection. Transduction was blocked by soluble YSA peptide and restored for EphA2-negative cells after recombinant EphA2 expression. The YSA peptide could also be inserted into three positions of a CAR binding-ablated HAdV-5 fiber enabling specific transduction; however, the Ad5T/41sSK format was superior *in vivo*. In conclusion, we establish an adenovirus capsid facilitating functional insertion of targeting peptides and a novel adenovirus using the tumor marker EphA2 as receptor with high potential for cancer gene therapy and viral oncolysis.

## Introduction

Recombinant adenoviruses (Ads) are in pre-clinical and clinical development as oncolytic agents and vectors for vaccination and gene therapy [1], [2].uch Ad-based drugs can considerably benefit from or even depend on a cell type-specific mode of action in order to limit side effects, while retaining therapeutic potency. Cell type-specificity of Ad-based vectors and oncolytics has been well established at the post-entry level by transcriptional targeting and microRNA regulation [3], [4].In contrast, targeting of Ad cell entry, which would better avoid side effects and virus sequestration, remains a challenge. Genetic strategies for Ad entry targeting facilitate simple clinical grade production (no chemical modifications or separate adapters are required) and ensure that progeny oncolytic Ads produced in the patients' tumors retain the improved properties. However, strategies for genetic entry targeting of Ads are hampered by the rigid Ad capsid structure, the inability of antibody molecules, as preferred targeting moieties, to fold properly after fusion to cytosolically expressed Ad capsid proteins, and the complex interactions of host factors modulating Ad cell entry in patients (see below).

Entry targeting of Ads requires two steps: ablation of native tropism for healthy cells and re-targeting of a receptor specifically expressed on target cells by insertion of a corresponding ligand into the Ad capsid. Cell entry of native Ads is initiated by binding of the capsid protein fiber to the attachment receptor, which is Coxsackie-Ad receptor (CAR) for the most widely used serotype HAdV-5 [5].Virus internalization is then triggered by binding of the penton base to cellular integrins [6].For therapeutic applications of Ads not intended to target hepatic tissue, liver de-targeting is of key importance to avoid toxic side effects. However, ablating CAR binding by mutating the fiber did not reduce liver transduction after systemic virus injection [7].Additional deletion of integrin binding by mutating the penton base resulted in reduced liver transduction in some but not all studies [7].Moreover, penton base mutations might be counterproductive in the context of Ad targeting, as virus entry and post-entry steps of re-targeted viruses can be compromised [8].Liver transduction by HAdV-5, irrespective of their interaction with CAR and integrins, was attributed to blood coagulation factors bound to hexon and fiber [9]–[11].Which receptor mediates transduction of hepatocytes by HAdV-5 *in vivo* is still being discussed [12], [13].

Our approach for entry targeting of HAdV-5-derived viruses is to replace the fiber shaft and knob domains with the corresponding domains of the HAdV-41 short fiber (Ad5T/41sSK) and to insert peptide ligands into this chimeric capsid. HAdV-41 binds CAR via a second long fiber, while no cell-binding activity has been attributed to the short fiber. Correspondingly, strongly reduced transduction *in vitro* and liver transduction *in vivo* have been demonstrated by several groups for HAdV-5-based vectors containing short fibers of HAdV-41 or of the closely related HAdV-40 [14]–[19].We have previously demonstrated that infectivity of Ads with chimeric Ad5T/41sSK fiber (then termed F5/41s) can be restored by genetic peptide ligand insertion using the integrin binding RGD4C-peptide as a model peptide [14].In fact, we identified several functional insertion sites, thus establishing the chimeric Ad5T/41sSK fiber as a flexible fiber scaffold for ligand insertion: the HI and EG loops on the side of the knob and for the IJ loop on its top, resulting in superior transduction efficiency compared with C-terminal fusions. However, as integrins are ubiquitously expressed, the RGD4C peptide was not suitable to demonstrate the potential of the Ad5T/41sSK format for cell type-specific cell entry and transduction. Therefore, the aim of the present study was to establish cell entry targeting by the Ad5T/41sSK strategy using a cell-selective peptide ligand and to compare this strategy with a HAdV-5 fiber-based targeting approach.

The YSA peptide, a 12-mer identified by phage display, selectively binds to the receptor tyrosine kinase EphA2, but not to related kinases [20].We focused our study on this peptide ligand because in contrast to several other tested peptides it retained cell-binding activity in the context of the Ad fiber. Importantly, EphA2 is gaining increasing attention as target for cancer therapy because it is (i) upregulated on most solid tumors and on tumor endothelium, (ii) better accessible on tumors that often lack cell-associated ligands, (iii) functionally associated with tumor progression, and (iv) was recently reported to be a cancer stem cell marker [21], [22].Several EphA2-targeted therapeutic modalities have shown proof of concept in pre-clinical studies, including kinase inhibitors, antibodies, immunotoxins, engineered T cells, soluble receptors, and vaccines [22]–[24].

Here, we investigated Ad entry targeting *in vitro* and *in vivo* by genetically inserting the EphA2-binding YSA peptide into different receptor-blind Ad fiber scaffolds. Specifically, we explored sites for functional YSA peptide insertion into the knob domains of the short HAdV-41 fiber and of the HAdV-5 fiber. In addition to virus production by combined fiber transfection/virus superinfection as we have done before [14],we investigated direct engineering of fiber genes in the virus genomes, which is of advantage or required for ease of virus manufacturing and for viral oncolysis, respectively. Selectivity and efficiency of Ad cell entry mediated by the YSA peptide was investigated in cell culture, human metastases biopsies, and animal xenograft models comparing three fiber formats: (i) the chimeric Ad5T/41sSK fiber, (ii) a long-shafted chimeric fiber containing the HAdV-5 fiber tail and shaft domains and the short HAdV-41 fiber knob, and (iii) a long-shafted but CAR-binding ablated HAdV-5 fiber.

## Results

### Specific transduction of EphA2-positive cells by Ads with YSA peptide inserted into chimeric fibers containing the knob of the HAdV-41 short fiber

**Figure 1:**
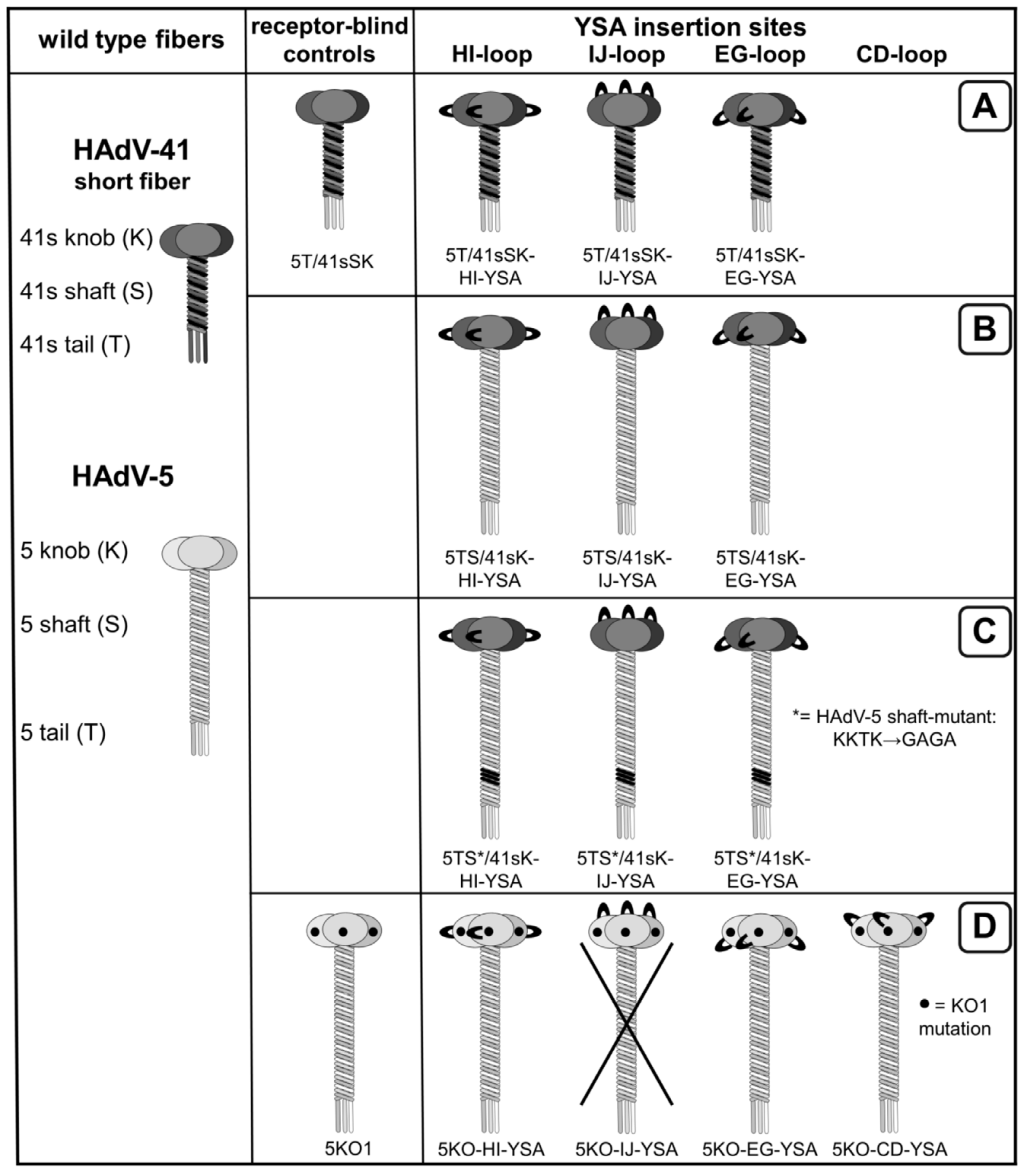
Schematic outline of fiber constructs with YSA peptide insertion investigated in this study. (**A**) Short-shafted chimeric fibers with the HAdV-5 tail fused to the shaft and knob of the HAdV-41 short fiber. (**B,C**) Long-shafted chimeric fibers containing the HAdV-5 tail and wild-type (B) or mutant (C) shaft fused to the HAdV-41 short fiber knob. (**D**) CAR binding-ablated HAdV-5 fibers. YSA peptide insertion sites are indicated by loops. Insertion of the linker sequence in different positions of the HAdV-5 fiber knob IJ loop resulted in loss of trimerization capacity (data not shown) and viruses were not produced for this insertion site.

**Figure 2:**
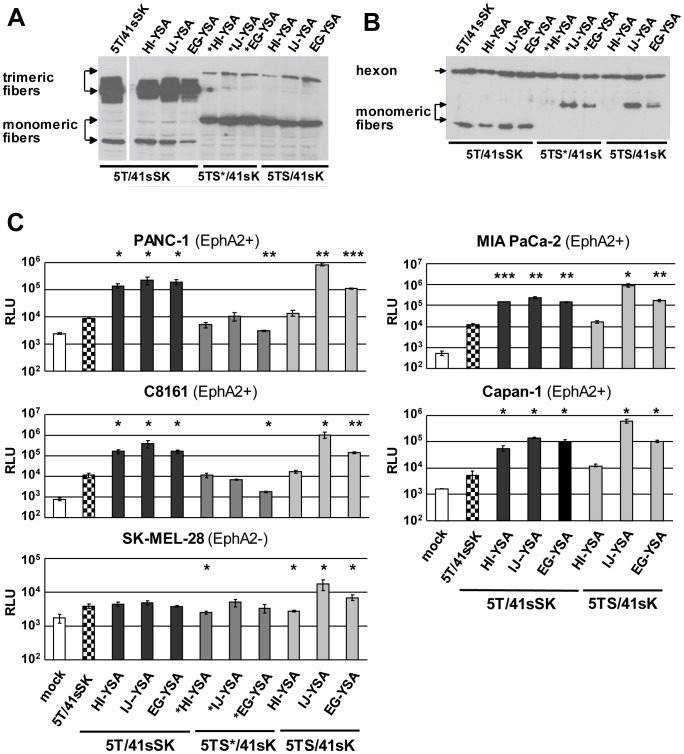
Long- and short-shafted chimeric fibers with genetic insertion of the YSA peptide into the HAdV-41 short fiber knob: trimerization, incorporation into virus particles, and viral transduction. (**A**) Immunoblot of unboiled cell lysates after transient transfection of fiber expression plasmids into 293T cells. (**B**) Immunoblot of purified virus particles of pseudotyped LacZ reporter viruses generated by the transfection/superinfection protocol. (**C**) Transduction of EphA2-positive and EphA2-negative cells with pseudotyped LacZ reporter viruses. SK-MEL-28 cells were transduced in quadruplicates, all other cell lines were transduced in triplicates. Columns and error bars show mean values and standard deviations of β-Gal activity, respectively. RLU, relative luminescence units; * p<0.05, ** p<0.01, *** p<0.001 versus 5T41sSK in the respective cell line.

We investigated entry targeting of Ads by genetic insertion of a targeting peptide into chimeric fibers with HAdV-41 knob as a de-targeted scaffold. To this end, we inserted the 12-mer EphA2-binding peptide YSA [20] flanked by short linkers into the HI, IJ or EG loops of this knob domain. To explore the relevance of shaft length on YSA-mediated Ad transduction, we combined these YSA-containing knobs with the short HAdV-41 fiber shaft (Ad5T/41sSK viruses, Fig. 1A) or the long HAdV-5 fiber shaft (Ad5TS/41sK viruses, Fig. 1B). In a third set of fibers, we incorporated the long HAdV-5 fiber shaft with a mutated heparin sulfate proteoglycan (HSPG)-binding motif (Ad5TS*/41sK viruses, Fig. 1C). This mutation was reported to confer improved de-targeting [17], [25], [26].After plasmid transfection, all constructs were expressed and possessed trimerization capacity, but trimerization was clearly less efficient for the long-shafted constructs, especially those containing the peptide in the HI loop (Fig. 2A). Using a combined transfection/superinfection protocol (see Materials and Methods), we were able to produce high titer pseudotyped LacZ reporter Ad vectors with all fiber formats. Incorporation of fiber molecules into viral particles was efficient for the short-shafted fiber and, despite reduced trimerization capacity, for the long-shafted IJ-YSA fibers (Fig. 2B). Long-shafted EG-YSA and HI-YSA fibers showed reduced or lacked fiber incorporation into virus particles, respectively.

Next, we analyzed EphA2 expression in a panel of pancreatic cancer cells, melanoma cells, endothelial cells, and a hepatic cell line. We detected strong EphA2 expression for pancreatic cancer cells, endothelial cells, and for 4 of 6 melanoma cell cultures (Fig. S1). EphA2 was not detected for the two melanoma cell lines SK-MEL-28 and Mel624 and the hepatic cell line HepG2. Transduction experiments with EphA2-positive cell lines PANC-1, MIA PaCa-2, Capan-1 (pancreatic cancer) and C8161 (melanoma) yielded results that were similar for the respective cell lines: All three viruses pseudotyped with the short-shafted YSA fibers showed transduction, which was substantially stronger than for the control viruses without YSA peptide (Fig. 2C; 10-fold to 35-fold increase in β-Gal activity). For viruses with unmodified HAdV-5 shaft, the insertion of YSA into the HI, IJ and EG loops showed weak, strong, and intermediate transduction efficiency, respectively. The long-shafted IJ-YSA virus was even superior to the short-shafted viruses (p<0.05 for all cell lines.). For all viruses with the mutated long shaft, we observed inefficient transduction (shown in PANC-1 and C8161 cells). In EphA2-negative SK-MEL-28 cells, the EG-YSA virus and especially the IJ-YSA virus with unmodified long shaft showed transduction significantly higher than the control viruses. We conclude that short-shafted Ad5T/41sSK viruses with YSA peptide inserted into the EG, HI or IJ loop selectively transduce EphA2-positive tumor cells, while the long shafted Ad5TS/41sK-IJ-YSA virus showed even stronger, but less selective transduction.

**Figure 3:**
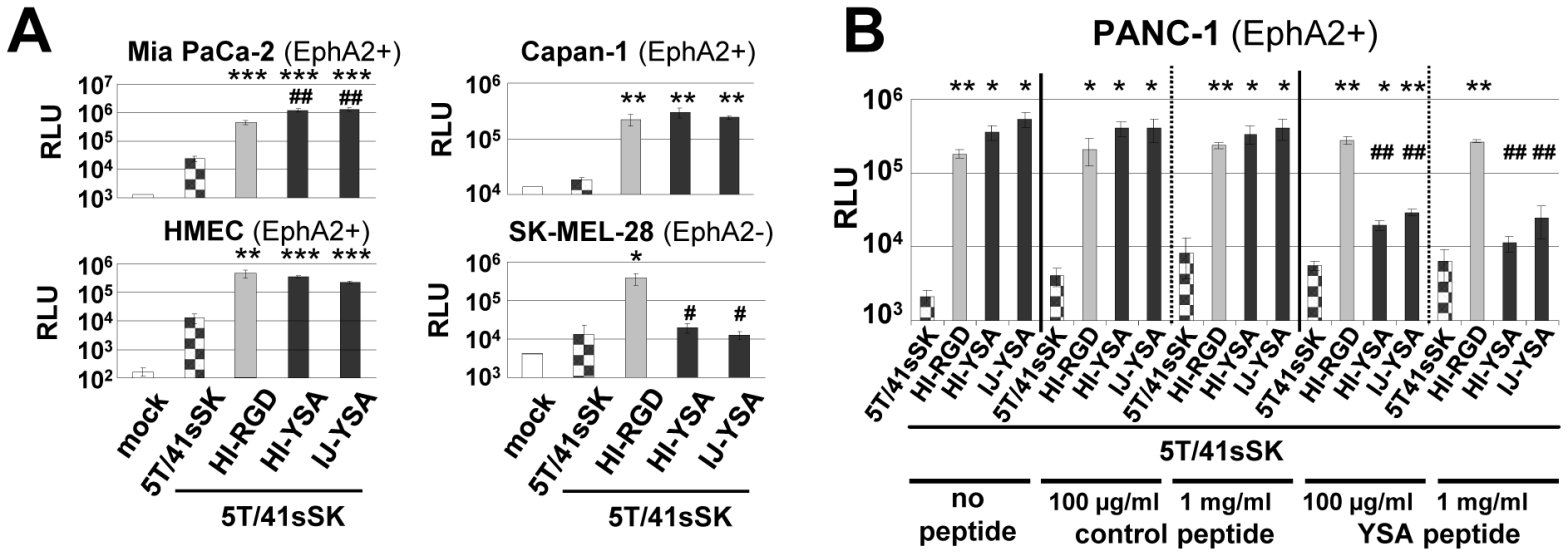
Efficiency and selectivity of transduction by Ad vectors containing short-shafted chimeric fibers with genetic insertion of the YSA peptide. (**A**) Transduction of EphA2-positive and EphA2-negative cells by pseudotyped Ad vectors containing short-shafted chimeric fibers with genetic insertion of the YSA peptide in comparison to matching vectors with genetic insertion of the RGD4C peptide. Cells were transduced in quadruplicates. (**B**) Transduction of EphA2-positive PANC-1 cells after competition with soluble YSA peptide or with control peptide of randomized sequence. Cells were transduced with pseudotyped Ads in triplicates. Columns and error bars show mean values and standard deviations of β-Gal activity, respectively. RLU, relative luminescence units; *^/#^p<0.05, **^/##^p<0.01 ***p<0.001 versus 5T/41sSK (*) or 5T/41sSK-HI-RGD (^#^).

Next, we investigated how the transduction efficiency of pseudotyped YSA viruses compares with viruses containing the established, integrin-binding RGD peptide in tumor and endothelial cells. Most previous studies on genetic peptide ligand insertion used RGD-containing peptides and demonstrated improved, but not targeted cell entry [27]–[29].Indeed, HAdV-5 viruses with RGD peptide in the HI loop are being investigated in clinical studies, because of their improved cell entry efficiency [30].We found that in the context of the Ad5T/41sSK chimeric fiber YSA peptide-mediated transduction of EphA2-positive cells was similar or even superior to transduction mediated by the RGD4C peptide inserted in the HI loop (Fig. 3A and B), the most effective insertion site for this peptide [14].In EphA2-negative SK-MEL-28 cells, only the RGD virus resulted in transduction significantly higher than the control virus without peptide. Finally, we could show that transduction of EphA2-positive cells by Ad5T/41sSK-HI-YSA and Ad5T/41sSK-IJ-YSA but not Ad5T/41sSK-HI-RGD is effectively blocked by soluble YSA peptide, while a control peptide with randomized sequence did not block transduction (Fig. 3B). Overall, these results demonstrate potent YSA-mediated transduction specifically of EphA2-positive cells by Ads with YSA peptide inserted into the chimeric Ad5T/41sSK fiber.

### Specific transduction of EphA2-positive cells by Ads with YSA peptide inserted into the CAR binding-ablated HAdV-5 fiber

**Figure 4:**
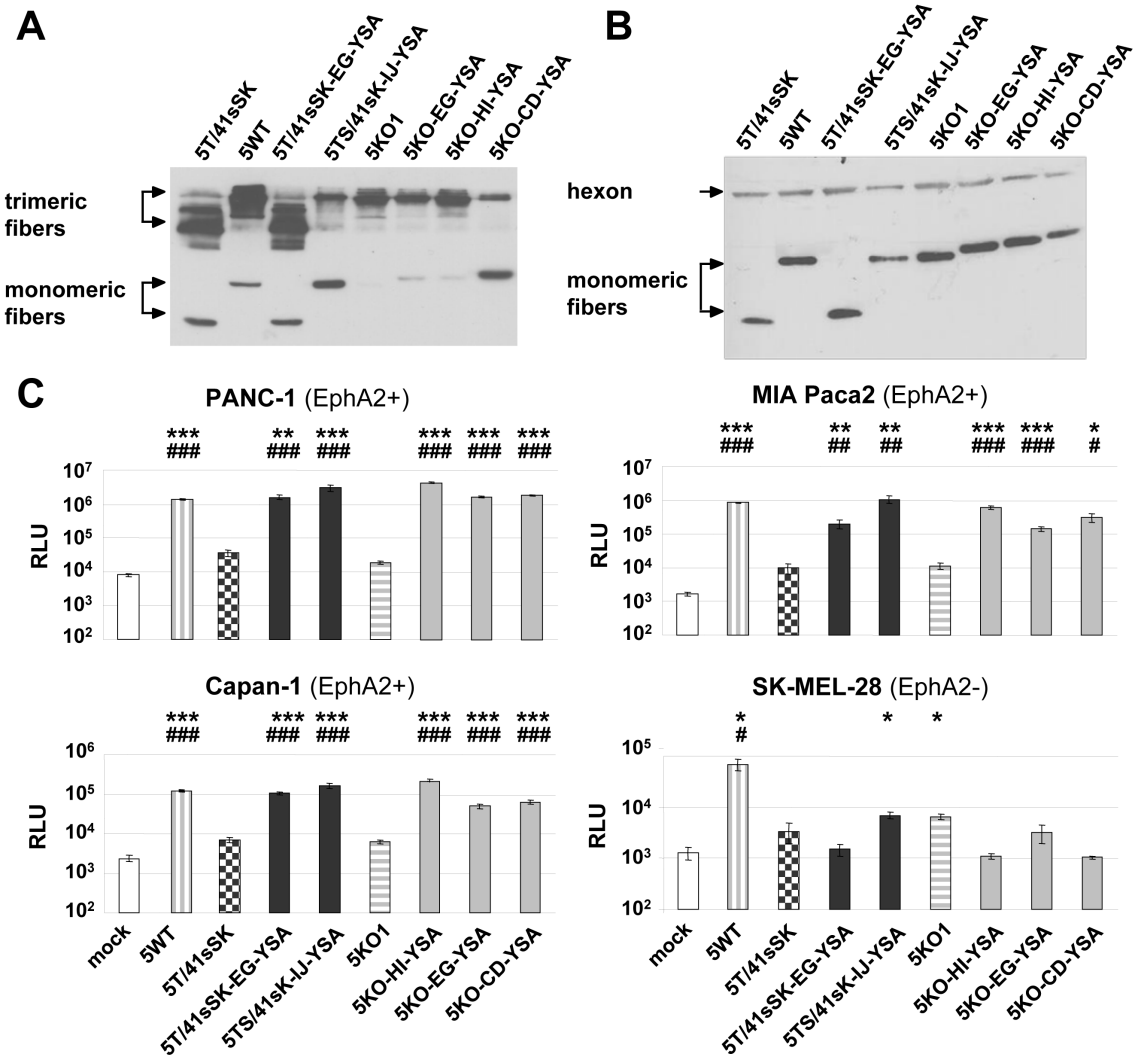
Fibers with genetic insertion of the YSA peptide into the CAR binding-ablated HAdV-5 knob: trimerization, incorporation into virus particles, and transduction. (**A**) Immunoblot of unboiled cell lysates after transient transfection of fiber expression plasmids into 293T cells. (**B**) Immunoblot of purified virus particles of pseudotyped LacZ reporter viruses. (**C**) Transduction of EphA2-positive and EphA2-negative cells with pseudotyped LacZ reporter viruses. Cells were transduced in quadruplicates, columns and error bars show mean values and standard deviations of β-Gal activity, respectively. RLU, relative luminescence units; *^/#^p<0.05, **^/##^p<0.01, ***^/###^p<0.001 versus 5T/41sSK (*) or 5KO1 (^#^).

For specific applications, e.g. those requiring tumor-specific gene transfer but not liver de-targeting (see discussion), Ads with a HAdV-5-based fiber might be sufficient or advantageous compared with more extensively modified chimeric fiber-containing viruses. For this reason and to compare our chimeric fiber format with the native one, we investigated which insertion sites enable genetic incorporation of the YSA peptide into the CAR binding-ablated HAdV-5 fiber knob KO1 (S408E and P409A mutations, Ref [31], Fig. 1D). We found that the YSA peptide can be inserted into the CD, EG and HI loops of the fiber retaining trimerization capacity (somewhat less efficient for the CD loop) and incorporation capability into virus particles generated by the transfection/superinfection protocol (Fig. 4A and B). We also explored the IJ loop as a possible position for peptide ligand insertion considering the favorable location on the top of the knob domain. However, we observed loss of fiber trimerization after inserting a linker at positions G560/H561 or I564/N565 (not shown). Pseudotyped Ads with the YSA peptide inserted into the EG, HI or CD loop of the KO1 knob mediated efficient transduction of EphA2-positive cells, which was 8-fold to 230-fold superior to the control virus Ad5KO1 without peptide insertion (Fig. 4C). The Ad5KO-HI-YSA virus was more efficient than the Ad5KO-EG-YSA and Ad5KO-CD-YSA viruses in all cell lines tested as well as superior to Ad5T/41sSK-EG-YSA. Furthermore, the Ad5KO-HI-YSA virus showed similar or superior transduction efficiency compared with the Ad5WT virus, containing the wild-type HAdV-5 fiber. In EphA2-negative cells, none of the viruses with YSA peptide inserted into the KO1 fiber increased transduction efficiency compared with the parental virus (Fig. 4C), demonstrating a lack of off-target transduction. In conclusion, the transduction efficiency of Ads with YSA peptide insertion into the HAdV-5 fiber depends on the insertion site with the HI insertion showing the best transduction efficiency and specificity.

### EphA2-mediated transduction of tumor and endothelial cells by genomically fiber-modified Ads

**Figure 5:**
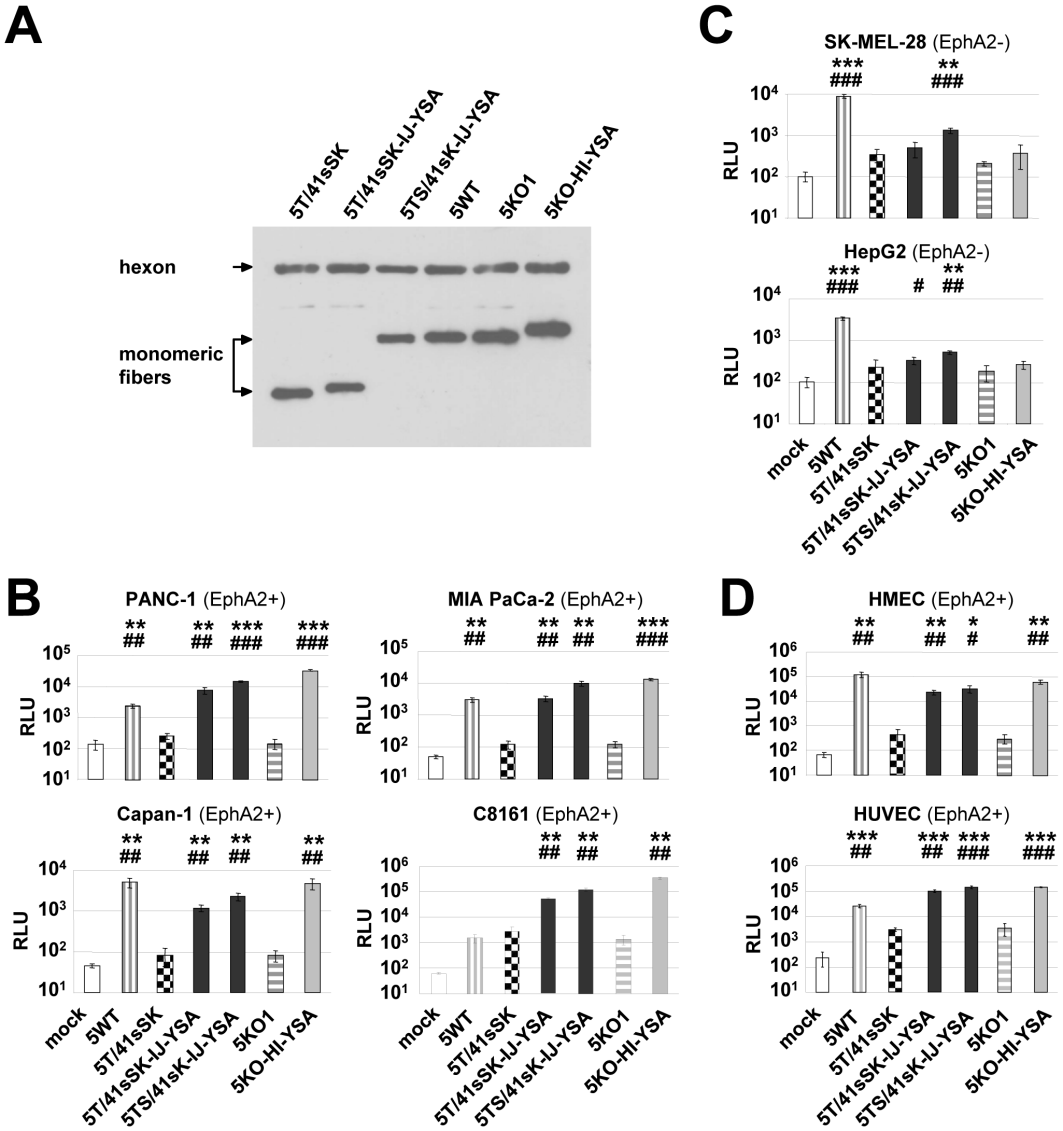
EphA2-targeted Ads with genomically inserted fibers containing the YSA peptide: incorporation of fibers into virus particles and transduction. (**A**) Immunoblot of purified virus particles generated by genomic insertion of recombinant fibers. (**B–D**) Transduction of EphA2-positive cancer cells (B), EphA2-negative cells (C) and EphA2-positive endothelial cells (D) with genomically fiber-modified Luc/GFP reporter viruses. C8161 cells were transduced in triplicates, all other cell lines were transduced in quadruplicates. Columns and error bars show mean values and standard deviations of Luc expression, respectively. RLU, relative luminescence units; *^/#^p<0.05, **^/##^p<0.01, ***^/###^p<0.001 versus 5T/41sSK (*) or 5KO1 (^#^).

**Figure 6:**
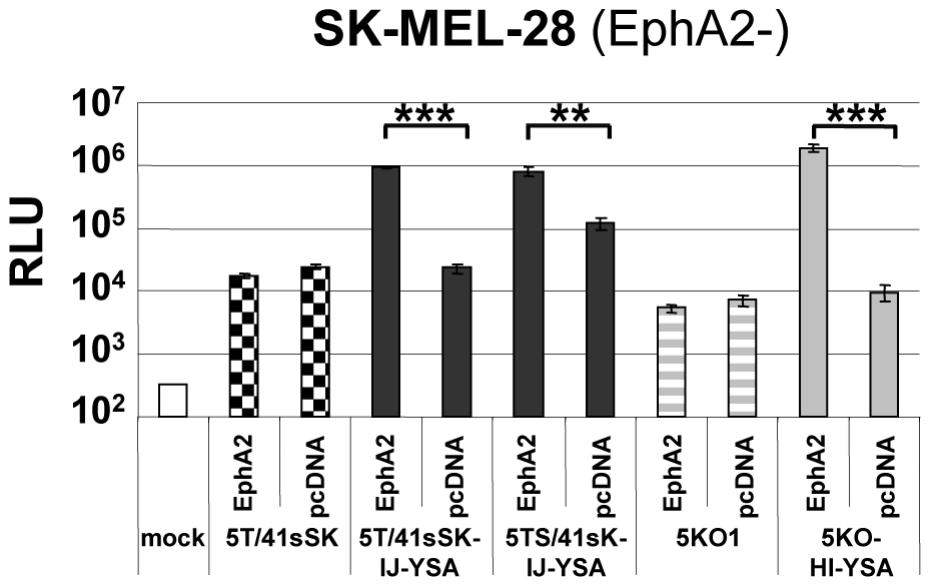
Transduction of EphA2-negative cells expressing recombinant EphA2 with YSA peptide-containing Ads. SK-MEL-28 cells transfected with EphA2 expression plasmid (pcDNA-EphA2) or control plasmid (pcDNA) were transduced with genomically fiber-modified Luc/GFP reporter viruses. Cells were transduced in quadruplicates, columns and error bars show mean values and standard deviations of Luc expression, respectively. RLU, relative luminescence units; ** p<0.01, ***p<0.001 for indicated comparisons. For detection of EphA2 expression of transfected cells see Fig. S3.

We next developed EphA2-targeted viruses with modified fibers encoded by their genome rather than pseudotyped by transfection. Genomically modified viruses would simplify manufacturing procedures and facilitate large scale production of viruses. Also, genomic fiber insertion is required in the context of viral oncolysis. However, it is not clear how genomic fiber replacement affects the production of infectious viruses. In fact, a previous study reported growth defects and/or defective particles for Ad vectors with the native fiber gene replaced by a gene encoding the entire short HAdV-41 fiber with peptide insertions [32].Using BAC recombineering technology, we cloned six genomes of first generation GFP/Luc reporter viruses. In three genomes the HAdV-5 fiber was replaced by a YSA-containing fiber representing each of the fiber formats (Ad5T/41sSK-IJ-YSA, Ad5TS/41sK-IJ-YSA and Ad5KO1-HI-YSA). The other three genomes represented the control viruses Ad5T/41sSK, Ad5wt, and Ad5KO1. All viruses could be produced at high titer and showed efficient fiber incorporation (Fig. 5A). Results of transduction experiments with these genetically modified viruses reproduced those obtained with pseudotyped viruses produced by the transfection/superinfection protocol, demonstrating that genomic fiber insertion was successful (Fig. 5B–D): In EphA2-positive pancreatic cancer cells, melanoma cells and endothelial cells, we again observed a dramatic increase in transduction efficiency for YSA-containing viruses compared with receptor-blind control viruses (15-fold to 236-fold). Insertion of the YSA peptide into the KO1 fiber resulted in the highest increase in transduction efficiency. The IJ-YSA fiber chimeric virus with long shaft was again somewhat stronger than the matching virus with a short shaft. Of note, YSA viruses resulted in stronger transduction even compared with the virus containing the wild-type HAdV-5 fiber in EphA2-positive cells. This was the case not only for cells weakly transduced with viruses containing the HAdV-5 fiber, i.e. C8161 cells (36-fold to 220-fold), but also in the EphA2- and CAR-positive [33], [34] cells MIA PaCa-2, PANC-1 and HUVEC. In EphA2-negative SK-MEL-28 and HepG2 cells, Ad5T/41sSK-IJ-YSA and Ad5KO1-HI-YSA showed no significant increase in transduction efficiency compared with the respective controls without peptide insertion, whereas Ad5TS/41sK-IJ-YSA again showed somewhat increased transduction. Visualization of GFP expression confirmed that Ads with YSA peptide resulted in transduction of markedly increased percentages of EphA2-positive cells compared with control viruses without peptide and also compared with Ad5wt (Fig. S2). In EphA2-negative cells, transduction efficiency as determined by this GFP-based read-out was strongly reduced for Ad5T/41sSK-IJ-YSA, Ad5KO1-HI-YSA, Ad5T/41sSK and Ad5KO1 in comparison to Ad5wt, but less so for Ad5TS/41sK-IJ-YSA. These results confirm that Ad5T/41sSK-IJ-YSA and Ad5KO1-HI-YSA viruses feature the best entry targeting capacity for EphA2-positive cells. Of note, transduction of SK-MEL-28 cells by YSA viruses but not by control viruses without peptide insertion could be restored by overexpression of recombinant EphA2 (Fig. 6, Fig. S3), demonstrating that EphA2 mediates cell entry of YSA peptide-containing viruses.

### YSA peptide-mediated adenoviral transduction of pancreatic cancer and melanoma xenografts *in vivo*

**Figure 7:**
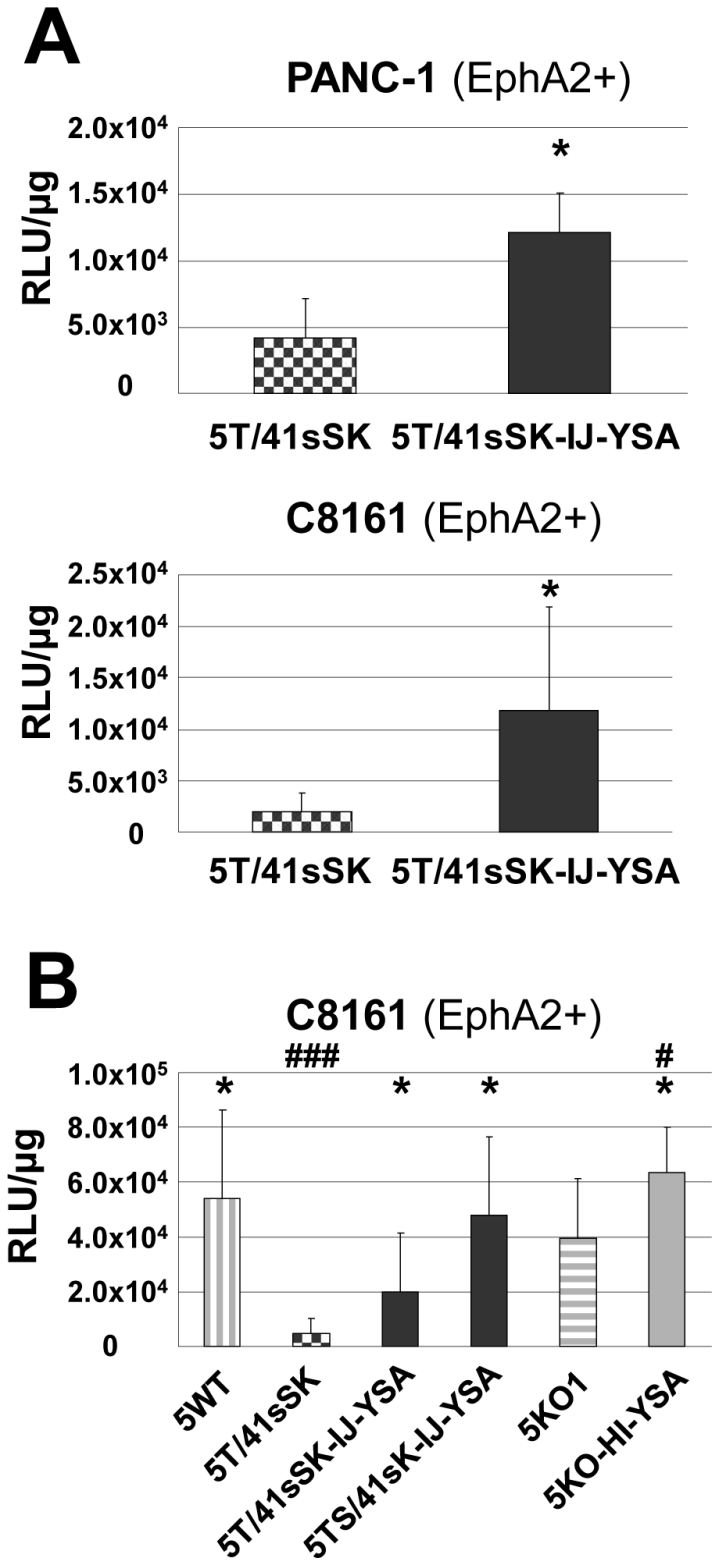
Transduction of EphA2-positive tumor xenografts *in vivo*. (**A,B**) 2×10^10^ vp of genomically fiber-modified, EphA2-targeted Luc/GFP reporter and control viruses were injected intratumorally into NOD-SCID mice carrying tumor xenografts (n = 6 to 9 tumors per group). Luciferase reporter gene expression in tumors was quantified 3 days after virus injection. Columns and error bars show mean values and standard deviations, respectively. RLU, relative luminescence units. * p<0.05 versus 5T/41sSK, ^#^p<0.05 and ^###^p<0.001 versus 5KO1.

Production of high titer preparations of genomically capsid-modified viruses allowed us to perform animal studies to explore YSA peptide-mediated adenoviral transduction *in vivo*. To this end, we used NOD-SCID mice with subcutaneous xenograft tumors of PANC-1 pancreatic cancer cells or C8161 melanoma cells. EphA2 expression *in vivo* was verified in both tumor models (Fig. S4). In a first experiment, we injected animals bearing PANC-1 or C8161 tumors intratumorally (i.t.) with Ad5T/41sSK-IJ-YSA or Ad5T/41sSK (Fig. 7A). In both tumor models we observed significantly stronger transduction with Ad5T/41sSK-IJ-YSA (2.9-fold for PANC-1 and 5.9-fold for C8161), demonstrating that YSA-peptide-mediated transduction is functional *in vivo*. In a second experiment we injected C8161 tumors i.t. with the YSA-containing Ads representing each of the fiber formats (Ad5T/41sSK-IJ-YSA, Ad5TS/41sK-IJ-YSA and Ad5KO1-HI-YSA) or with control viruses (Fig. 7B). Increased transduction for Ad5T/41sSK-IJ-YSA versus Ad5T/41sSK was confirmed. Ad5TS/41sK-IJ-YSA showed somewhat higher transduction than Ad5T/41sSK-IJ-YSA. These results are in accord with the *in vitro* transduction results. However, in contrast to the *in vitro* data Ad5KO1 showed similar transduction efficiency than Ad5wt after i.t. injection into C8161 xenografts revealing loss of de-targeting. Accordingly, the increase in transduction by YSA peptide insertion was minor (1.6-fold), but reached significance. Therefore, de- and re-targeting of Ads for specific entry via EphA2 after i.t. injection *in vivo* was best implemented with the Ad5T/41sSK fiber format. Overall, these results show that peptide-mediated entry targeting using the Ad5T/41sSK fiber format is functional *in vivo* after i.t. virus injection.

### YSA peptide-mediated adenoviral transduction in biopsies of human melanoma metastases

**Figure 8:**
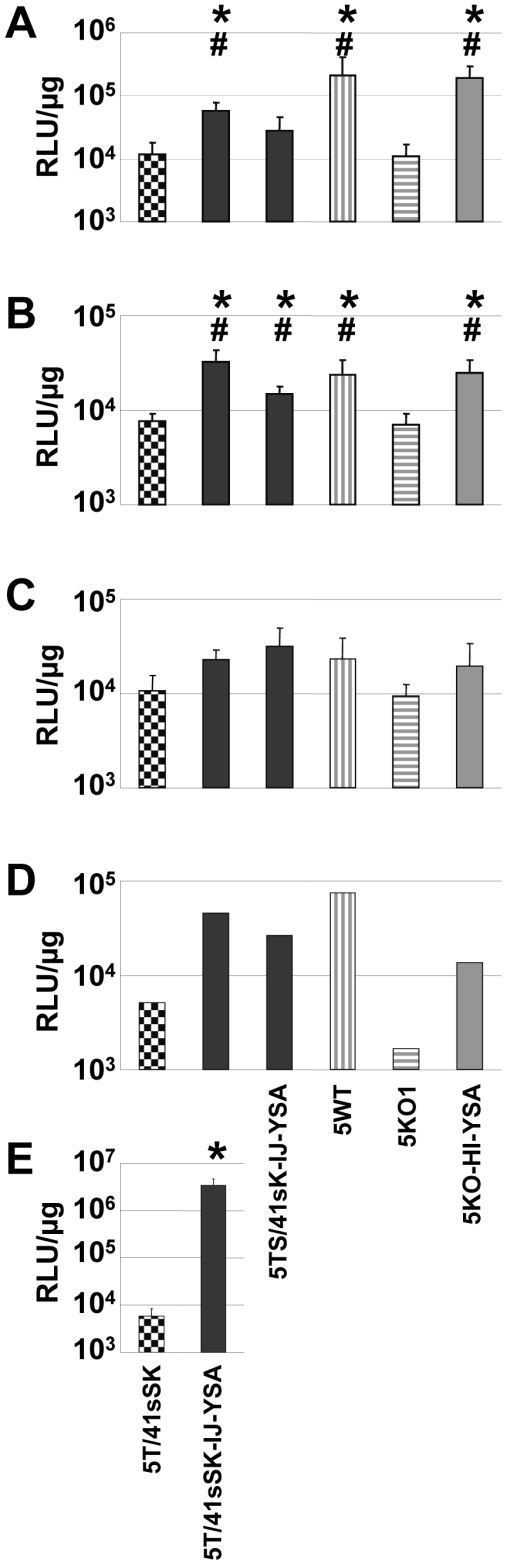
Transduction of living tissue slices of human melanoma metastases *ex vivo*. (**A–E**) Living tissue slices of melanoma metastases from 5 different patients were transduced with 10^10^ vp/slice of genomically fiber-modified, EphA2-targeted Luc/GFP reporter and control viruses. The number of transduced slices per virus was dependent on the size of the biopsy and was n = 4 (A,B), n = 3 (C), n = 2 (D) and n = 5 (E). Luciferase reporter gene expression was quantified 3 days post-transduction. Columns (A–E) and error bars (A,B,C,E) show mean values and standard deviations, respectively. RLU, relative luminescence units. *^/#^p<0.05 versus 5T/41sSK (*) or 5KO1 (^#^).

We next investigated whether EphA2-targeted Ads result in increased transduction not only in monolayer tumor cell cultures and tumor cell xenografts, but also in freshly biopsied tumor material from patients. These represent clinically more relevant substrates with respect to tumor cell physiology and presence of tumor microenvironment. Biopsies of melanoma metastases were cut into living tissue slices immediately after surgery and were subsequently transduced with genomically capsid-modified, YSA-containing Ads representing each of the fiber formats or with control viruses. EphA2 expression was detected in living tissue slices of all biopsies obtained from 5 patients. However, expression varied between patients and was weaker than for monolayer cultures (Fig. S5A). This was expected as biopsies contain a mixture of cells of which only a fraction are tumor cells. We observed strongest transduction efficiency as determined by GFP expression for viruses with YSA peptide and for the control virus with native HAdV-5 fiber, confirming YSA peptide-mediated transduction (Fig. S5B). As GFP signals in 2D photographs do not quantitatively represent transduction efficiency, in part due to the wrinkled shape of the slices after 3 day culture, we quantified luciferase expression for these slices (Fig. 8A) and for biopsies from four additional patients (Fig. 8B-E, one biopsy yielded only enough slices for transduction with Ad5T/41sSK-IJ-YSA and Ad5T/41sSK). Note that luciferase readings are high because we performed high titer transductions to enable monitoring of GFP expression. We observed a markedly increased transduction for Ad5T/41sSK-IJ-YSA compared with Ad5T/41sSK in 5 of 5 biopsies (significance was reached in 3 biopsies with 4.3- to 576-fold differences, not in the remaining biopsies due to limited material). The strongest increase in transduction was observed for the biopsy that showed strongest EphA2 expression. Ad5TS/41sK-IJ-YSA showed higher transduction than Ad5T/41sSK in 4 of 4 biopsies, but was lower than for Ad5T/41sSK-IJ-YSA in 3 of 4 biopsies, which was in contrast to results in monolayer cultures. Finally, transduction of melanoma living tissue slices by Ad5KO1-HI-YSA was strongly increased compared with Ad5KO1 in 4 of 4 biopsies (2.1-fold to 17.1-fold, significant in 2 biopsies). These results confirm re-targeted adenoviral transduction mediated by the inserted YSA peptide for Ad5T/41sSK-IJ-YSA and Ad5KO1-HI-YSA also in clinically relevant tumor biopsy material.

## Discussion

In this study we establish cell type-specific Ad cell entry by functional peptide ligand insertion into a de-targeted chimeric fiber scaffold containing the short HAdV-41 fiber shaft and knob (Ad5T/41sSK). Moreover, we describe Ad vectors entry-targeted to the highly relevant pan-cancer surface marker EphA2 by genomic insertion of the gene encoding the chimeric fiber or a CAR binding-ablated HAdV-5 fiber with YSA peptide ligand. Transduction of EphA2-positive cells *in vitro* was dramatically increased by peptide ligand insertion for both fiber formats (up to 236-fold), underlining the potency of the YSA peptide for re-targeting of viral cell binding and entry. We previously identified the EG, HI and IJ loops of Ad5T/41sSK as sites for functional insertion of the RGD4C peptide, which binds to widely expressed integrins [14]. Here we confirmed the feasibility of these insertion sites using the EphA2-binding YSA peptide. In contrast to the RGD peptide, which showed superior activity in the HI loop, we observed similar activity in each of the three loops for the YSA peptide (Fig. 2), revealing that insertion sites affect peptide-receptor interactions differently dependent on the peptide. With the YSA peptide, we could demonstrate for the first time that the chimeric Ad5T/41sSK fiber facilitates cell type-specific Ad transduction using a panel of EphA2-positive and EphA2-negative cells (Figs. 2 and 5). Peptide competition and recombinant receptor expression studies clearly prove that transduction is peptide-specific and mediated by EphA2 (Figs. 3 and 6). These results establish a rationale for future studies that should explore whether further ligand peptides are functional in the Ad5T/41sSK fiber scaffold, enabling virus entry targeting via other surface markers.

We demonstrate that the introduction of the modified fibers into the virus genome, thereby replacing the native fiber, is feasible (Fig. 5) in addition to pseudotyping via the two step transfection/superinfection protocol, as also used in our previous study ([14], Figs. 2–4). We observed efficient fiber trimerization, virus production and fiber incorporation into virus particles for the genomically modified viruses with 5T/41sSK fibers (and KO1 fibers, see below). A previous study showed for genomically fiber-modified viruses expression, but no incorporation of HAdV-41 short fibers with peptide inserted into the AB, CD, or HI loops, or G region when a second HAdV-7 fiber was present [32]. Of note, both the specific insertion positions and the inserted peptides differed from those in our study. Furthermore, the fiber tail domain was derived from HAdV-41, which we previously reported to result in reduced fiber trimerization and incorporation when compared with the native HAdV-5 tail used by us [14]. Still, in the Schoggins study the short HAdV-41 fiber without peptide insertion was preferentially incorporated into viral particles in presence of the HAdV-7 fiber indicating that the peptide insertions affected fiber incorporation [32]. We conclude that the EG, HI and IJ loops of the HAdV-41 short fiber knob are recommended for insertion of ligand peptides, establishing a panel of fiber scaffolds for this purpose. However, fiber trimerization and incorporation into virus particles need to be evaluated individually for each ligand. The Schoggins paper also reports maturation and cell entry defects for virus particles containing the HAdV-41 short fibers with insertions in the G region. Note that these viruses differ from our viruses also by deletion of the penton RGD motif, which might contribute to virus cell entry defects. Furthermore, maturation defects were dependent on the sequence of the inserted peptide. Although we did not explicitly investigate virus particle maturation, we were successful in producing high titer viruses that show efficient fiber incorporation and transduction similar or even superior to matching viruses containing the HAdV-5 fiber (Fig. 5). These results argue that the Ad5T/41sSK fiber scaffold described here and in our previous study [14] is compatible with genomic fiber modification, facilitating the development of entry-targeted Ad vectors at high quantity and quality and of entry-targeted oncolytic Ads *per se*.

Our study further demonstrates that entry targeting is not enhanced by replacement of the short HAdV-41-derived shaft in the chimeric Ad5T/41sSK fiber with the long HAdV-5 shaft, a strategy we pursued hypothesizing that ligand-receptor interactions would be improved. In fact, we observed reduced fiber trimerization for chimeric viruses with long shaft (5TS/41sSK and 5TS*/41sSK constructs, Fig. 2), indicating that trimerization is hampered when fusing the HAdV-41 short fiber knob with the HAdV-5 shaft. As only trimeric fibers can be incorporated into viral particles, is it somewhat surprising that Ad5TS*/41sSK and Ad5TS/41sSK fibers with YSA peptide inserted into the IJ loop show normal fiber content in purified viruses (Figs. 2 and 5). This result shows that even low-level trimerization can be sufficient to ensure fiber incorporation. However, incorporation of fibers into viral particles was reduced or lost after YSA peptide insertion into the EG or HI loops, respectively (Fig. 2). Trimerization and incorporation deficiency of the Ad5TS/41sSK-HI-YSA fiber is in contrast to results for the HAdV-5 fiber, which is susceptible to YSA peptide insertion into the HI loop (Figs. 4, 5 and [53], [54]). This difference can be explained by the different sequence, length and intramolecular environment of the HI loops in the HAdV-41 short fiber versus the HAdV-5 fiber. However, note that short shafted HAdV-41 fibers accepted YSA insertions into the HI loop without loss of trimerization and incorporation (Figs. 2 and 5), because of the above mentioned critical role of the shaft domain for fiber trimerization and incorporation into virus particles. Ad5TS/41sK viruses with YSA peptide inserted into the EG loop and especially into the IJ loop resulted in significant transduction of EphA2-negative cells. This property might be independent of the inserted ligand, as Nakamura et al. previously reported increased Ad transduction *in vitro* and *in vivo* for the HAdV-40 short fiber knob when fused to a long fiber shaft [16]. Possible explanations are direct cell binding via the shaft domain [26] or shaft length-dependent modification of cell binding properties of the knob. The latter was shown for CAR-binding knobs, which mediate strongly reduced adenoviral transduction when fused to a short shaft [16]. Finally, YSA-mediated viral transduction was lost irrespective of the peptide insertion site, when a HAdV-5 fiber shaft containing a mutation of the putative HSPG binding motif (KKTK to GAGA) was used (Fig. 2), which is in accord with previous studies [14], [35]–[38] and is hypothesized to result from reduced shaft flexibility and/or defective post-entry virus trafficking.

The chimeric Ad5T/41sSK fiber represents a promising novel scaffold for peptide ligand insertion, especially considering its reported reduced biodistribution to the liver after i.v. injection [14], [15], [17]–[19]. However, its suitability for applications in the context of oncolytic Ads, which depend on full replication potency, remains to be demonstrated. For specific applications requiring tumor-specific activity but not liver-detargeting, we considered the CAR binding-ablated HAdV-5 fiber as candidate scaffold suitable for ligand insertion. We show in this study that functional YSA peptide insertion is feasible in the CD, FG and HI loops with the HI loop giving the best results (Fig. 4). However, the CD and FG insertion sites might still be suitable for other peptide ligands, since our data for the chimeric Ad5T/41sSK fiber show that insertion site preferences depend on the inserted peptide. Several previous studies showed functional peptide ligand insertion into the HI loop or fusion to the C-terminus of the HAdV-5 fiber [39]. Moreover, Ads with RGD peptide insertion into the HI loop, featuring enhanced infectivity, are being investigated in clinical gene therapy and virotherapy studies [30]. Internal insertion sites other than the HI loop have been rarely studied, but should be considered in the future. Transduction by Ad5KO-HI-YSA was slightly more effective and selective compared with Ad5T/41sSK-IJ-YSA *in vitro* (Figs. 4 and 5). Thus, both virus formats warrant further investigation in gene therapy and virotherapy applications.

In xenograft experiments, we found that i.t. virus injection resulted in strongly reduced tumor transduction for Ad5T/41sSK when compared with Ad5WT, while this was not the case for Ad5KO1. Of note, this was observed also for xenografts of CAR-negative C8161 cells (Fig. 7). These results point at CAR-independent tumor transduction *in vivo* mediated by HAdV-5 fibers, a conclusion also supported by a previous study reporting that CAR binding ablation does not de-target HAdV-5 vectors after i.t. injection [37]. Thus, we show that Ad5T/41sSK possesses superior de-targeting features after i.t. application, while CAR binding-ablated HAdV-5 fibers require further optimization for efficient de-targeting. Importantly, YSA peptide insertion into the Ad5T/41sSK fiber resulted in a significant increase in the transduction of EphA2-positive pancreatic cancer and melanoma xenografts, indicating peptide-mediated viral entry targeting *in vivo* (Fig. 7). Ad5KO-HI-YSA viruses showed a minor but significant increase in tumor transduction compared with Ad5KO1 (Fig. 7). Peptide-mediated increase in adenoviral transduction after i.t. injection was frequently shown for Ads with RGD peptide genetically inserted into the HAdV-5 HI loop [28], [40].However, RGD-containing viruses also mediate increased transduction of healthy tissues, as the targeted integrins are widely expressed [29].Inconsistent results were reported for i.t. injection of viruses with other genetically inserted peptide ligands that mediate targeted transduction *in vitro*. Some studies reported lack of peptide mediated transduction after i.t. injection in the context of CAR-binding[41] or CAR binding-ablated HAdV-5 [37].Other studies reported increased transduction (3-fold) or selective oncolytic activity after i.t. injection of Ads with genetically inserted peptide in the HAdV-5 fiber binding or not binding CAR, respectively [42], [43].

Our i.t. injection data show for the YSA peptide that the affinity was sufficient to mediate targeted viral cell entry *in vivo*. Future studies will need to investigate whether this strategy of genetic ligand peptide insertion into the Ad5T/41sSK fiber mediates targeted transduction after systemic adenovirus injection. Previous reports showed that peptide insertion into the HAdV-5 HI loop, even when mediating effective transduction *in vitro*, did not necessarily target virus transduction after systemic application [37], [44].Thus, entry-targeted Ads or their delivery mode might require further improvement in order to overcome additional barriers to tumor homing after systemic injection. We believe that attempts to reduce the interaction with host factors that neutralize or sequester virus particles, such as antibodies and blood coagulation factors [12],or strategies to overcome anatomical barriers, such as vessel walls and tumor matrix [45]–[47] are warranted. Strategies that have been reported but remain to be investigated in combination with entry-targeted viruses include additional capsid modifications [48],virus shielding [49],increased tumor blood vessel permeabilization [46],carrier cells [50],matrix degradation [51],and cell junction openers [52].

EphA2 attracts increasing attention as a target for cancer therapy [22] and therefore represents a promising target for tumor-delivery of therapeutic viruses. This is because EphA2 is overexpressed in many tumors, including tumor stem cells, and functionally associated with tumor progression [22]. Importantly, a high affinity peptide ligand for EphA2 is available, the YSA peptide. Our study demonstrates functional genetic insertion of the YSA peptide into the Ad capsid and shows entry targeting *in vitro* and *in vivo* with best results for the chimeric Ad5T/41sSK fiber format. Two previous studies by van Geer *et al.* support the feasibility of the YSA peptide for entry targeting of Ad transduction *in vitro* [53], [54]. In these studies, the YSA peptide was inserted into the HI loop of the HAdV-5 fiber either still able to bind CAR [54] or de-targeted from both CAR and integrins [53]. The latter virus, however, was strongly attenuated compared with a matching virus containing the wild-type HAdV-5 fiber. In contrast, Ad5KO1-HI-YSA reported in our study possesses similar or even superior activity to Ad5WT in the same EphA2-positive cell lines used by van Geer and colleagues (Figs. 4 and 5), indicating that either the KO1 backbone is superior or a secondary penton base-integrin interaction supports EphA2-mediated Ad cell entry. Van Geer *et al.* reported a lack of EphA2-mediated adenoviral tumor transduction *in vivo* when injecting their double-ablated YSA Ads i.v. or i.p. into mice with xenografts of pancreatic cancer cells [53]. However, they did not perform i.t. injections, which in our study were successful to demonstrate peptide-mediated entry targeting *in vivo*.

In conclusion, we developed a capsid-modified Ad vector, Ad5T/41sSK-IJ-YSA, targeted to cancer cells *in vitro* and *in vivo* by using the pan-cancer marker EphA2 as receptor for virus entry. Ad5T/41sSK-IJ-YSA offers interesting opportunities for applications in gene therapy and virotherapy of several cancers reported to overexpress EphA2. Moreover, the chimeric fiber scaffold of Ad5T/41sSK might be exploited for targeting other tumor markers by insertion of corresponding ligands.

## Materials and Methods

### Ethics statement

All animal experimental procedures were approved by the responsible Animal Protection Officer at the German Cancer Research Center and by the regional authorities (Regierungspräsidium Karlsruhe), according to the German Animal Protection Law. Biopsies of human melanoma metastases were obtained from the Department of Dermatology, University Hospital Heidelberg, Germany, with written informed consent from the donor according to the Declaration of Helsinki Principles and with approval of the local Ethics Committee (Ethikkommission Medizinische Fakultät Heidelberg, Votum S-337/2008).

### Cell culture

Human cell lines 293 (Q-Biogene, Montreal, Canada), 293T (ATCC), 633 [55], A375M [56], C8161 [57], Capan-1 (ATCC), HepG2 (ATCC), HMEC-1 (Life Technologies, Darmstadt, Germany), Mel624 [58], Mel888 [58], MiaPaCa-2 (ATCC), PANC-1 (ATCC), HUVEC (PromoCell, Heidelberg, Germany) and low passage human melanoma cells purified from human melanoma metastasis biopsies, PMelL, were cultivated using standard cell culture conditions as described before [14].

### Fiber expression plasmids

Fiber expression plasmids were cloned based on pDV67 (kindly provided by Glen Nemerow, La Jolla, CA [55];). Plasmids encoding chimeric fibers 5T/41sSK and 5TS*/41sK with cloning site for ligand insertion were previously described [14]. 5TS/41sK constructs were obtained by re-mutation. The sequence encoding the YSAYPDSVPMMS-peptide (Ref [20]) was inserted by oligonucleotide cloning (see Materials and Methods S1 for listing of oligonucleotides). The pKO1 expression plasmid encoding a CAR binding-ablated HAdV-5 fiber (S408E and P409A mutations, ref [31]) was generated by site-directed mutagenesis. A GGSGG linker containing a BamHI-site was inserted into pKO1 by site-directed mutagenesis between (i) G450/T451 in the CD-loop; (ii) K468/S469 in the EG-loop; (iii) T542/G543 in the HI-loop; (iv) G560/H561 in the IJ-loop or (v) I564/N565, also in the IJ-loop. The YSA-peptide was inserted by oligonucleotide cloning as described above. All fiber expression plasmids were sequenced.

### Transient transfection assays

For analysis of fiber trimerization, 5.0×10^5^ 293T cells per well were seeded in 3 ml of medium containing 2% FBS in 6-well plates. Cells were transfected the next day with 5 µg of fiber expression plasmid per well, using Lipofectamine (Life Technologies, Darmstadt, Germany) according to the manufacturer's instructions. 72 h after transfection, cells were washed in PBS and lysed in 100 µl of 100 mM Tris by gently rocking for 1 h at 4°C. 3 µl of 5 M NaCl were added, cell debris was pelleted and supernatants were analyzed by immunoblot. For transient EphA2 expression, 2.5×10^4^ SK-MEL-28 cells per well were seeded in 48-well plates. The next day cells were transfected in quadruplicates with 1.5 µg per well of EphA2 expression plasmid pcDNA4-EphA2mychis [59] or of control plasmid without insert using Lipofectamine. After 24 h cells were transduced with Ads and processed for reporter gene analysis. In parallel, cell lysates of transfected cells were analyzed for EphA2 expression by immunoblotting.

### Immunoblotting

30 µg boiled or unboiled lysates from cell cultures, living tissue slices, or homogenized tumors or 5.0×10^9^ boiled viral particles were separated by SDS-PAGE and transferred to a nitrocellulose membrane (Schleicher und Schuell, Dassel, Germany). Membranes were probed with a mouse monoclonal antibody binding to the HAdV-5 fiber tail (clone 4D2; Abcam, Cambridge, UK) or with a mouse monoclonal antibody specific for EphA2 (clone D7; Millipore, Darmstadt, Germany). As loading control, membranes were probed with a mouse monoclonal antibody specific for β-actin (clone AC-74; Sigma, Deisenhofen, Germany) or with rabbit monoclonal antibody binding to HAdV-5 hexon (ab24240; Abcam). Bound antibodies were detected with an anti-mouse-HRP (#7076S; Cell Signaling, Danvers, MA, USA) or with an anti-rabbit-HRP (#7074; Cell Signaling) secondary antibody followed by enhanced chemiluminescence (Thermo Scientific, Schwerte, Germany).

### Generation of LacZ reporter Ads pseudotyped with modified fibers by a two step transfection/superinfection protocol

Genomically fiberless LacZ reporter viruses were phenotypically equipped (pseudotyped) with a fiber by transient transfection of 293T cells with the respective fiber expression plasmid followed by superinfection with Ad5.βGal.ΔF (kindly provided by AdVec Inc., Ontario, Canada and Glen Nemerow, La Jolla, CA) as described by Hesse *et al.* [14] Viruses were harvested 48 h post-infection and purified by CsCl equilibrium density gradient ultracentrifugation. Genomic integrity was verified by PCR, restriction digests, and sequencing. Medium virus yields were between 2000 and 8900 vp/cell for viruses with HAdV-41 short fiber knob and between 900 and 1400 vp/cell for viruses with HAdV-5 KO1 mutant fiber knob.

### Generation of genomically fiber modified reporter Ads expressing GFP and luciferase by BAC-recombineering

For a detailed protocol of BAC recombineering see Refs [60] and [61], which our protocol was adapted from. First, the genome of an E1/E3-deleted GFP and luciferase reporter virus (QBiogene, Irvine, CA, USA) was provided with the BAC-backbone pAd5-FRT (kindly provided by Zsolt Ruzsics, Munich, Germany [62],). The latter was amplified by PCR using primers with a 5′-overhang of 30 nucleotides corresponding to both ends of the viral genome to allow homologous recombination in SW102 bacteria (kindly provided by Stephen P. Creekmore, NCI, Frederick, MD). Second, also by homologous recombination, the fiber gene coding sequence was replaced by the sequence of a GalK/Amp selection cassette which was amplified from plasmid pT GalK-Amp (kindly provided by Stephen P. Creekmore) using primers with a 5′-overhang of 30 nucleotides corresponding to the flanking position in the Ad genome were the fiber gene should be removed. Third, again by homologous recombination, the cassette was exchanged by the fiber sequence of interest amplified from the cloned expression plasmids. Details about the cloning procedures can be obtained upon request. Ad genomes were released from BACs by Pac I digest and were transfected into 293 cells using Lipofectamine. Viral particles were amplified on 293 cells. Viruses were harvested 48 h post-infection and purified by CsCl equilibrium density gradient ultracentrifugation. Genomic integrity was verified by PCR, restriction digests, and sequencing. Medium virus yields were between 9700 and 16300 vp/cell for all viruses.

### Virus quantification

Physical particle concentrations (vp/ml) of virus preparations were determined by OD_260_ determination and were used for transduction studies, which is standard procedure when comparing viruses with different capsids. Infectious virus titers described as 50% tissue culture infectious dose (TCID_50_) were additionally determined for viruses with genomic modification to ensure the quality of virus preparations. Similar vp-to-TCID_50_ ratios confirmed that the quality of virus preparations was not reduced by insertion of peptide ligand into the chimeric or KO1 fibers (Ad5wt: 13; Ad5T/41sSK: 58; Ad5T/41sSK-IJ-YSA: 57; Ad5TS/41sK-IJ-YSA: 53; Ad5KO1: 653; Ad5KO1-HI-YSA: 439).

### Adenoviral gene transfer assays

Cells were seeded in 48-well plates at a concentration of 2.5×10^4^ cells per well in 0.5 ml of medium containing 2% FBS. The following day, cells were pre-cooled by incubating the plates on ice for 20 min prior to transduction. Medium was removed from cell layers and the cells were transduced in triplicates or quadruplicates with Ads in 150 µl pre-cooled media containing 2% FBS. Transductions were performed using vp titers, as viruses compared in individual experiments possessed different capsid modifications that affect biological titers. For viruses generated by the two step transfection/superinfection protocol, 1×10^5^ vp/cell were used. For the genomically modified viruses 100 vp/cell (PANC-1, C8161, HMEC) or 200 vp/cell (Capan-1, MIA Paca-2, SK-MEL-28, HUVEC, HepG2) were used. After incubation on ice for 75 min, viruses were removed, cells were washed and cultivated in media containing 2% FBS. Cell-virus incubations were performed on ice to reduce non-specific viral uptake into cells under experimental conditions were high virus titers were used. Cells were harvested for reporter assay at 2 days post infection. Experiments were repeated at least once with the same virus preparation and were confirmed with an independent virus preparation. For peptide competition experiments with soluble 12mer YSA peptide or control peptide with randomized sequence (both PSL, Heidelberg, Germany) cells were preincubated for 20 min prior to transduction on ice with viruses in peptide-containing media.

### Reporter gene assays

Quantification of β-Gal activity was done using the Tropix kit (Life Technologies) according to the manufacturer's instructions. 50 µl lysate from each well was measured for 5 s using a Bertold luminometer. For quantification of Luc activity, cells were lysed in 150 µl cell culture lysis buffer (Promega, Mannheim, Germany) and frozen once. Living tissue slices and tumor homogenate were lysed in cell culture lysis buffer followed by three cycles of freezing and thawing. After centrifugation, the protein concentration in the supernatant was determined using the Bio-Rad DC™ Protein Assay kit (München, Germany). Luciferase activity was quantified using 50 µl lysate and 5 sec measurement in a Berthold luminometer using luciferin substrate solution [63].

### *In vivo* gene transfer

5×10^6^ C8161 or PANC-1 cells were implanted s.c. into both flanks of 6- to 8-week-old female NOD/SCID mice (in-house breeding, German Cancer Research Center). On an average volume of 200 mm^3^, 2×10^10^ vp in 100 µl PBS were injected i.t. into both tumors. After 72 h, mice were sacrificed by cervical dislocation and tumors were extracted, snap frozen, and homogenized with a Micro Dismembrator.

### Living tissue slices

Tissues were sliced as previously described [64]. Slices were transduced with 1.0×10^10^ vp/slice for 75 min and kept at 37°C in a humidified atmosphere of 5% CO_2_ for 72 h.

### Statistical analysis

Differences between indicated groups were analyzed using the Student's *t* test. P values of <0.05 were considered statistically significant.

## Supporting Information

Figure S1 **EphA2 expression in a panel of pancreatic cancer, melanoma, hepatoma, and endothelial cells.** Detection of EphA2 expression in cell cultures by immunoblot. β-actin was used as loading control. Longer exposure is shown for upper right panel. Pancreatic cancer cell lines: PANC-1, Capan-1, and MIA Paca-2; melanoma cells: C8161, Mel888, PMelL, A375M, SK-MEL-28, and Mel624; endothelial cells: HUVEC (primary), and HMEC (immortalized); hepatoma cell line: HepG2.
(PDF)

Figure S2 **EphA2-targeted Ads with genomically inserted fibers containing the YSA peptide: transduction efficiency.** Transduction of EphA2-positive PANC-1 and C8161 cells and of EphA2-negative SK-MEL-28 cells with genomically fiber-modified Luc/GFP reporter viruses at 1000 vp/cell. GFP expression was visualized 48 h post-transduction (4x magnification).
(PDF)

Figure S3 **Transduction of EphA2-negative cells expressing recombinant EphA2 with YSA peptide-containing Ads: Detection of EphA2 expression.** EphA2 expression in SK-MEL-28 cells of the experiment depicted in Fig. 6, as detected by immunoblot. Cells were transfected with EphA2 expression plasmid (pcDNA-EphA2) or control plasmid (pcDNA). Lysate of C8161 cells was used as positive control. β-actin served as loading control.
(PDF)

Figure S4 **Transduction of EphA2-positive tumor xenografts** ***in vivo*** **: Detection of EphA2 expression.** Detection of EphA2 expression in tumor xenografts of the experiments shown in the indicated subfigures of Fig. 7 by immunoblot. Corresponding cell cultures and β-actin served as control.
(PDF)

Figure S5 **Transduction of living tissue slices of human melanoma metastases** ***ex vivo*** **.** Living tissue slices were transduced with 10^10^ vp/slice of genomically fiber-modified, EphA2-targeted Luc/GFP reporter and control viruses. (**A**) Detection of EphA2 expression in living tissue slices of the experiments shown in Fig. 8 by immunoblot. Lysate of C8161 cells was used as positive control and β-actin served as loading control. (**B**) Lower panels show GFP-expression of representative slices from the experiment presented in Fig. 8A at 3 days post-transduction (4x magnification). Upper panels show bright field pictures of the same slices.
(PDF)

Materials and Methods S1 **Supplementary materials and methods.**
(DOCX)
