# Supplementary figures and images for: Adenoviruses Using the Cancer Marker EphA2 as a Receptor In Vitro and In Vivo by Genetic Ligand Insertion into Different Capsid Scaffolds
Source: PLoS One. 2014 Apr 23;9(4):e95723. doi: 10.1371/journal.pone.0095723 (PMC3997477; doi:10.1371/journal.pone.0095723)

Fig. S1

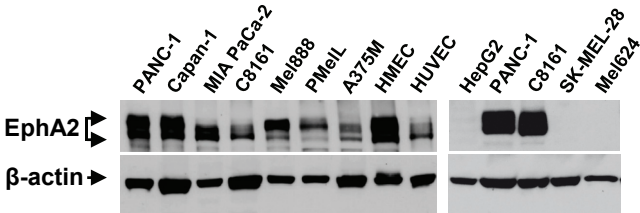

Supplement: Figure S1 — EphA2 expression in a panel of pancreatic cancer, melanoma, hepatoma, and endothelial cells. Detection of EphA2 expression in cell cultures by immunoblot. β-actin was used as loading control. Longer exposure is shown for upper right panel. Pancreatic cancer cell lines: PANC-1, Capan-1, and MIA Paca-2; melanoma cells: C8161, Mel888, PMelL, A375M, SK-MEL-28, and Mel624; endothelial cells: HUVEC (primary), and HMEC (immortalized); hepatoma cell line: HepG2. (PDF) [file pone.0095723.s001.pdf]

Fig. S2

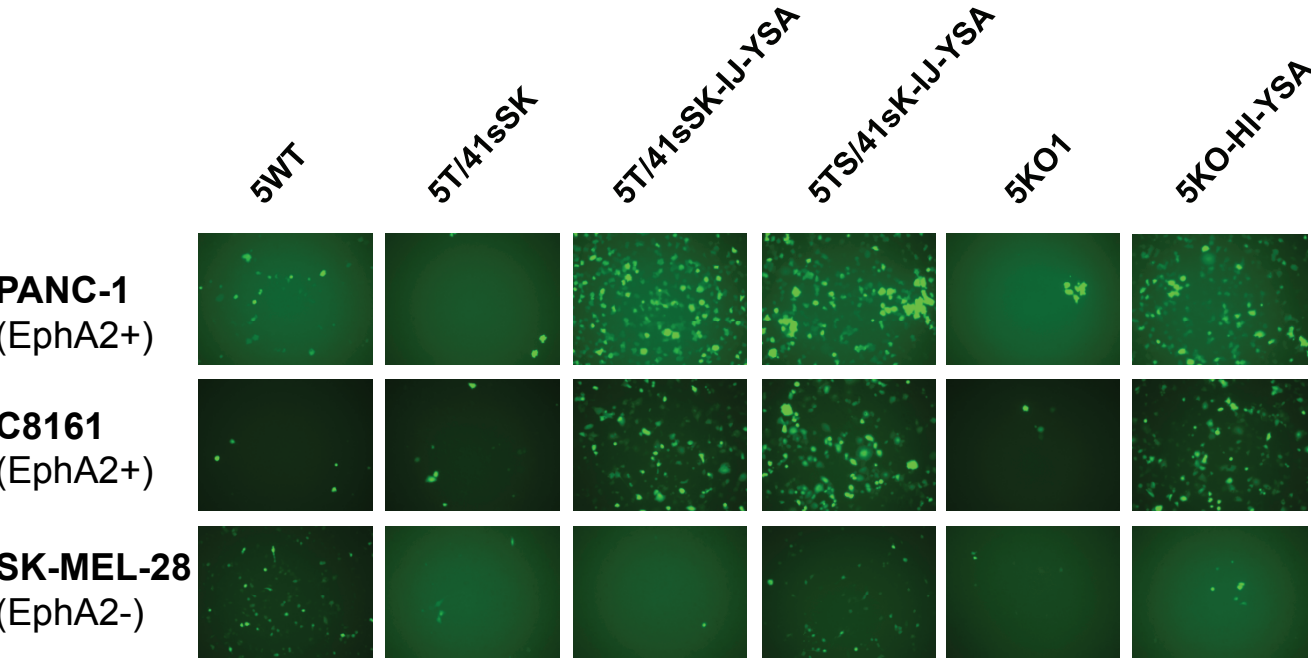

Supplement: Figure S2 — EphA2-targeted Ads with genomically inserted fibers containing the YSA peptide: transduction efficiency. Transduction of EphA2-positive PANC-1 and C8161 cells and of EphA2-negative SK-MEL-28 cells with genomically fiber-modified Luc/GFP reporter viruses at 1000 vp/cell. GFP expression was visualized 48 h post-transduction (4x magnification). (PDF) [file pone.0095723.s002.pdf]

**Fig. S3**

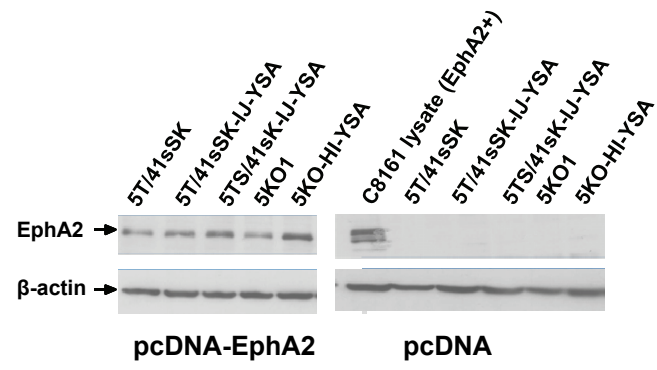

Supplement: Figure S3 — Transduction of EphA2-negative cells expressing recombinant EphA2 with YSA peptide-containing Ads: Detection of EphA2 expression. EphA2 expression in SK-MEL-28 cells of the experiment depicted in Fig. 6, as detected by immunoblot. Cells were transfected with EphA2 expression plasmid (pcDNA-EphA2) or control plasmid (pcDNA). Lysate of C8161 cells was used as positive control. β-actin served as loading control. (PDF) [file pone.0095723.s003.pdf]

**Fig. S4**

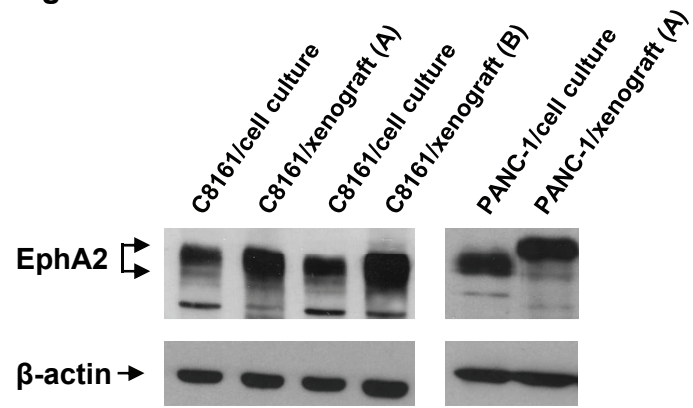

Supplement: Figure S4 — Transduction of EphA2-positive tumor xenografts in vivo : Detection of EphA2 expression. Detection of EphA2 expression in tumor xenografts of the experiments shown in the indicated subfigures of Fig. 7 by immunoblot. Corresponding cell cultures and β-actin served as control. (PDF) [file pone.0095723.s004.pdf]

Fig. S5

**A**

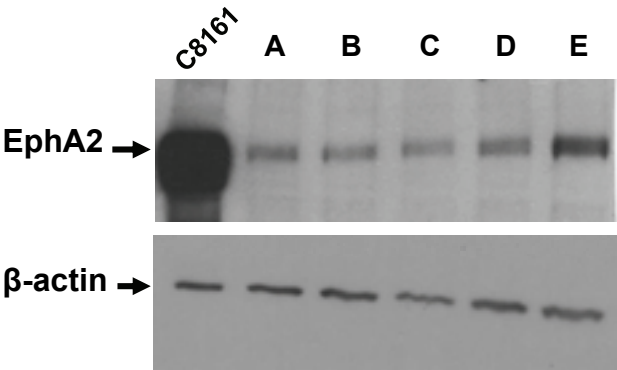

**B**

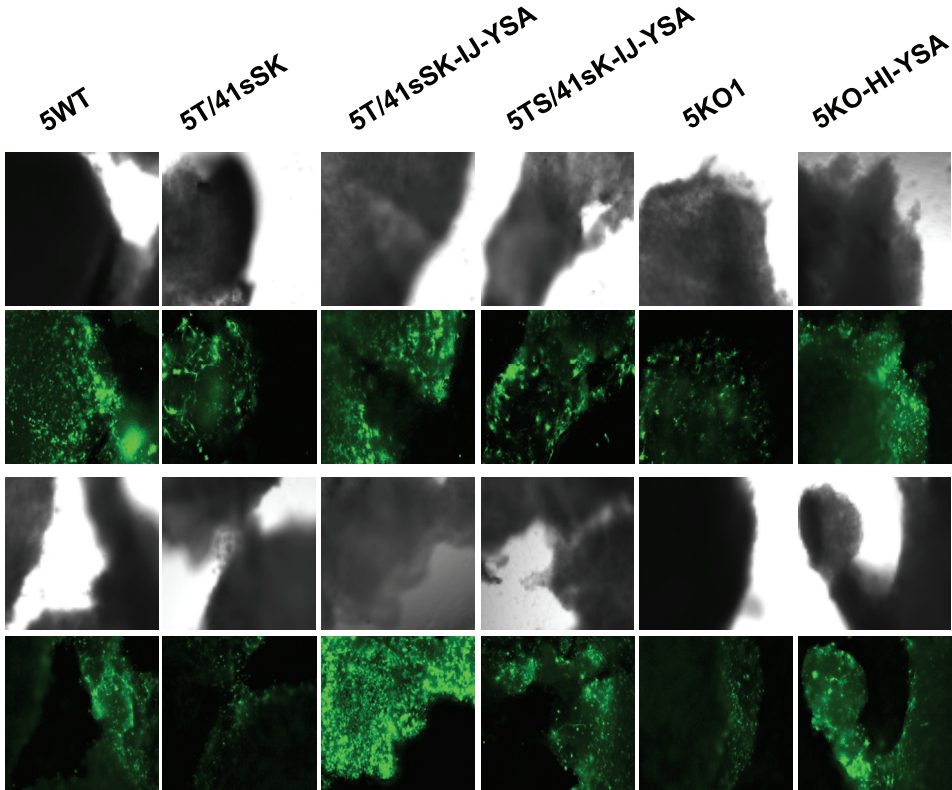

Supplement: Figure S5 — Transduction of living tissue slices of human melanoma metastases ex vivo . Living tissue slices were transduced with 1010 vp/slice of genomically fiber-modified, EphA2-targeted Luc/GFP reporter and control viruses. (A) Detection of EphA2 expression in living tissue slices of the experiments shown in Fig. 8 by immunoblot. Lysate of C8161 cells was used as positive control and β-actin served as loading control. (B) Lower panels show GFP-expression of representative slices from the experiment presented in Fig. 8A at 3 days post-transduction (4x magnification). Upper panels show bright field pictures of the same slices. (PDF) [file pone.0095723.s005.pdf]
