# Supplementary material for: Adenoviruses Using the Cancer Marker EphA2 as a Receptor In Vitro and In Vivo by Genetic Ligand Insertion into Different Capsid Scaffolds
Source: PLoS One. 2014 Apr 23;9(4):e95723. doi: 10.1371/journal.pone.0095723 (PMC3997477; doi:10.1371/journal.pone.0095723)
Supplement: Materials and Methods S1 — Supplementary materials and methods. (DOCX) [file pone.0095723.s006.docx]

Behr *et al.*

**Supporting Information Materials and Methods**

**Oligonucleotides for cloning of fiber expression plasmids**

Insertion of YSAYPDSVPMMS-peptide: 5’-GATCGTACTCCG-CCTACCCCGACTCCGTGCCCATGATGT-CCG-3’ and 5’-GATCCGGACATCATGGGC-ACGGAGTCGGGGTAGGCGGAGTAC-3’. Generation of pKO1 by site-directed mutagenesis: pKO1-fw 5´-ACCACACC-AGCTCCAGAGGCTAACTGTAGACTAAATGC-3´ and pKO1-rev 5´-GCATTTAGTCTA-CAGTTAGCCTCTGGAGCTGGTGTGGT-3´. Insertion of GGSGG linker containing a BamHI-site into pKO1 by site-directed mutagenesis: (i) in the CD-loop (KO-CD-L-fw: 5´-AGGATCCGGAGGGA-CAGTTCAAAGTGCTCATCT-3´ and KO-CD-L-rev: 5´-CGGATCCTCCTCCAGATATTG-GAGCCAAACTG-3´); (ii) in the EG-loop (KO-EG-L-fw: 5´-AGGATCCG-GAGGGTCTCACGGTAAAACTGCCAAAAG-3´ and KO-EG-L-rev: 5´-CGGATCCTCCT-TTTGGATAAGCTGATAGGTTAGG-3´); (iii) in the HI-loop (KO-HI-L-fw: 5´-AG-GATCCGGAGGGGGAGACACAACTCCAAGTGCATAC-3´ and KO-HI-L-rev: 5´-CGGA-TCCTCCTGTTTCCTGTGTACCGTTTAGTG-3´); (iv) G560/H561 in the IJ-loop (KO-IJ1-L-fw: 5´-AGGATCCGGAGGGCACAACTACATTAATGAAATATTTGC-3´ and KO-IJ1-L-rev: 5´-CGGATCCTCCGCCAGACCAGTCCCATGAAAATG-3´) and (v) I564/N565 in the IJ-loop (KO-IJ2-L-fw: 5´-AGGATCCGGAGGGAATGAAATATTTGCCACATCCTC-3´ and KO-IJ2-L-rev: 5´-CGG-ATCCTCCAATGTAGTTGTGGCCAGACC-3´).

**Detection of GFP expression.**

Fluorescence images were taken at 4x magnification using the Keyence BZ-9000 microscope to detect GFP expression.
